# Beyond K48 and K63: non-canonical protein ubiquitination

**DOI:** 10.1186/s11658-020-00245-6

**Published:** 2021-01-05

**Authors:** Michal Tracz, Wojciech Bialek

**Affiliations:** Faculty of Biotechnology, University of Wroclaw, Wroclaw, Poland

**Keywords:** Ubiquitin, Non-canonical, Atypical ubiquitination, Ubiquitin chains

## Abstract

Protein ubiquitination has become one of the most extensively studied post-translational modifications. Originally discovered as a critical element in highly regulated proteolysis, ubiquitination is now regarded as essential for many other cellular processes. This results from the unique features of ubiquitin (Ub) and its ability to form various homo- and heterotypic linkage types involving one of the seven different lysine residues or the free amino group located at its N-terminus. While K48- and K63-linked chains are broadly covered in the literature, the other types of chains assembled through K6, K11, K27, K29, and K33 residues deserve equal attention in the light of the latest discoveries. Here, we provide a concise summary of recent advances in the field of these poorly understood Ub linkages and their possible roles in vivo.

## Introduction

Protein ubiquitination (interchangeably called ubiquitylation) involves the covalent attachment of the 76-amino acid eukaryotic molecule ubiquitin (Ub) to substrate proteins. An enzymatic cascade of a Ub activating enzyme (E1), Ub-conjugating enzymes (E2s), and Ub ligases (E3s) governs the process, which is now recognized as an essential post-translational protein modification (PTM) of eukaryotic cells [1]. Ubiquitination is initiated by E1, an enzyme requiring ATP for the activation of Ub, which forms a thioester bond between the C-terminal Gly carboxyl group of Ub and its active site Cys. Upon activation, Ub is transferred onto the Cys residue of the E2 enzyme and substrate specificity is provided by the final enzyme in the cascade, the RING (really interesting new gene) or HECT (homologous to the E6-AP C-terminus)-type E3 ligase. As a result, isopeptide linkages are formed between the C-terminal carboxyl group of a Ub moiety and an ε-NH_2_ group on a lysine on the protein substrate or a preceding Ub moiety. In fact, E2-E3 complexes can decorate substrates with either a single Ub or several single Ub moieties (mono-multi-ubiquitination), or short Ub chains (oligoubiquitination). Alternatively, modification of the N-terminus or one of the seven lysine residues (K6, K11, K27, K29, K33, K48, and K63) of a Ub moiety already attached to the target protein leads to the elongation of Ub chains. Modification of the same or a different residue results in the formation of homogeneous or heterogeneous chains, respectively [2]. These multifaceted Ub chains—often referred to as the ‘ubiquitin code’—propagate specific cellular signals that we highlight in this brief review.

The ability of Ub to create different types of chains via its lysine residues represents one of the most powerful features of this protein. Each type of polyubiquitin chain has the potential to act as a distinct intracellular signal that must be specifically identified and decoded by proteins harboring Ub-binding domains (UBDs) to facilitate the diverse outcomes of ubiquitination within cells. The best-studied modification remains the canonical signal for degradation by the ubiquitin–proteasome system (UPS) where ubiquitinated substrates are recognized, bound, and degraded by the 26S proteasome, a 2.5-MDa cytosolic protein complex found in eukaryotic cells. This subject has been extensively explored in the literature over the last years and adequately reviewed [3–5]. The signal for degradation is created between the C-terminal residue of one Ub moiety and K48 of the previously conjugated Ub moiety [6]. In the process of protein degradation, deubiquitinases (DUBs) mediate the detachment of Ub chains from the protein to recycle Ub monomers for the next round of ubiquitination. Beyond their recycling role, DUBs are now widely recognized to coordinate proteasomal processing steps and modulate substrate degradation, imposing a checkpoint for proteasomal processing (recently reviewed in [7]).

K63-linked ubiquitination is the second best-known type [8], and an excellent review regarding linear ubiquitination is also available [9]. Notably, the implication of the former in endocytosis and the innate immune response, and the latter in the regulation of NF-κB signaling clearly demonstrate the role of ubiquitination in non-proteolytic cellular processes. This is in agreement with recent advances in the field of Ub biology indicating that protein degradation can no longer be regarded as the only possible outcome of ubiquitination. Currently, modification of substrate activities, modulation of protein localization or interactions, DNA damage repair, signal transduction, endocytosis, transcriptional regulation, and cell-cycle progression, have all been demonstrated as outcomes of ubiquitination [1]. In order to fulfill such diverse roles within cells, target proteins are modified by different types of Ub chains, which can now be more readily analyzed with the advent of powerful methods [10]. Unfortunately, understanding of atypical Ub linkages is still rather limited. Here, we briefly review the body of related work that is available on their roles.

## Lysine 6—autophagy and the DNA damage response

Much of the early work centered around the study of K6-linked ubiquitination in mitophagy, the autophagic processing of damaged mitochondria [11–14]. Depolarization of mitochondria, a symptom of their dysfunction, results in the embedment of PINK1 (PTEN-induced kinase (1) into the outer mitochondrial membrane (OMM) [15] where it phosphorylates Ub moieties attached to OMM proteins [12], as well as Parkin E3 ligase [16]. Once activated, Parkin decorates damaged OMM proteins with K6, K11, K48 and K63-linked chains [12] designating mitochondria for mitophagy [17]. This process is mainly promoted by K6 and K63-linked chains [11] but can be counteracted by Ub-specific protease USP8 that removes K6-linked chains from Parkin and prevents its autoubiquitination [13, 18]. Another DUB, USP30, the only one that is anchored into the OMM [19], also antagonizes Parkin-mediated ubiquitination and shows a clear preference for deubiquitination of K6-linked polyUb chains, even though it is able to remove other types of Ub linkages as well [14]. It has been suggested that USP30 regulates mitophagy by controlling the ubiquitination of many OMM proteins, including TOM20 [19]. As defective mitophagy is linked to Parkinson’s disease, USP30-specific inhibitors are suggested to be potentially beneficial for patients by regulating the balance between mitochondrial ubiquitination and deubiquitination in neurodegenerative disorders [20].

The DNA damage response (DDR) has been repeatedly shown to be controlled by K6-linked ubiquitination [21–25]. Pioneering studies reported K6-linked auto-ubiquitination of BRCA1-BARD1, a major DDR complex [21], and demonstrated the formation of K6-linked chains during replication stress and double-strand break repair [22, 26]. A much more recent line of evidence for DDR leading to this atypical ubiquitination stems from the research on HUWE1, an E3 ligase that generates the majority of cellular K6-linked species upon inhibition of the valosin-containing protein (VCP/p97/Cdc48) [24, 27]. VCP seems to mediate the disposal of proteins specifically targeted by HUWE1 [28], and HUWE1-generated K6-linked chains can pose a proteasomal degradation signal as shown for Mfn2 (mitochondrial fusogenic protein 2) [27]. Similarly to Parkin, HUWE1 and two other human E3 ligases, RNF144A (RING finger protein 144) and RNF144B, can assemble K6-, K11-, K48-, K63-linked chains in vitro [27]. A deeper understanding of this linkage combination is still required to elucidate its possible roles in the cellular context.

Further support implicating K6-linked Ub chains in protein degradation has been provided by the work on Viperin. This potent antiviral effector is targeted by virus-induced acetyltransferase HAT1 and UBE4A Ub ligase [29]. K197 of Viperin is first acetylated by the former enzyme to enable binding of UBE4A, which in turn stimulates ubiquitination with K6-linked chains at K206 and Viperin’s degradation [29]. Therefore, the antiviral immune response is substantially controlled by UBE4A because of its ability to regulate both virus- and IFN-induced Viperin protein production. It is worth noting that UBE4A is a mammalian homolog of yeast Ufd2 (see below), both belonging to a newly-discovered group of E3/E4 ligases which are thought to recognize and extend already assembled Ub species irrespective of their conjugates [30].

Contrary to their degradative roles, accumulated evidence indicates that K6-linked chains can be involved in protein stabilization [31–33] and other non-degradative processes [34, 35], highlighting the functional multiplexity of this atypical ubiquitination. The most recent and perhaps the most intriguing function of K6-linked polyubiquitination is as a DNA-binding enhancer during the innate immune system response [36]. Upon viral infection, the transcription factor IRF3 (interferon regulatory factor 3) is modified with K6-linked conjugates by an unknown E3 ligase. This enhances antiviral innate immunity, as only K6-linked ubiquitinated IRF3 can interact with the promoter of genes encoding type I interferons (IFN). Their transcription is negatively controlled by OTUD1, as upon OTUD1-mediated deubiquitination of IRF3 this transcription factor dissociates from target genome DNA [36]. Nevertheless, the mechanism of action governing the regulatory function of K6-linked ubiquitination on IRF3 is not yet understood.

## Lysine 11—not just another degradation signal

Despite the fact that K11-linked chains play a role in DDR [37], this atypical ubiquitination is generally associated with regulation of the cell cycle and proteasomal degradation. APC/C (Anaphase promoting complex/cyclosome) is a multi-subunit E3 RING ligase orchestrating mitosis and meiosis [38] and attaching K11/K48 mixed chains to substrates destined for proteasomal turnover [39, 40]. As a common motif with many other E3s, different E2s dictate the formation of various Ub chains [41, 42]. In the case of APC/C, its cooperation with UBE2C/UbcH10 and UBE2D/UbcH5 leads to the build-up of K48-linked chains, whereas UBE2S/APC/C interaction generates six to seven moieties-long K11-linked branch-offs [40, 42, 43]. Further studies demonstrated that UBE2S and APC/C are the major contributors to the K11-linked polyUb synthesis during mitosis [44]. Cells depleted of UbcH10 and UBE2S show impaired APC/C activity, leading to the stabilization of all APC/C substrates tested [45].

K11-linked chains are often accompanied by their K48 counterparts. Either of these homotypic conjugates can initiate protein degradation on their own [46], but their mutual incorporation, combined with chain branching, strongly enhances recognition by proteasomal receptor subunits in yeast [40, 47]. This may be in part ascribed to a conformation attained by the branched trimer, in which a novel hydrophobic interface between the distal Ub is found [47]. In humans, K11/K48-branched chains assembled by UBR4 and UBR5 provide proteasome-dependent quality control of mitotic regulators, misfolded nascent polypeptides and pathological Huntingtin variants [48–50]. In *Drosophila*, the Ci family of transcription factors involved in Hedgehog signaling is modified with K11/K48-linked chains to control their activation and repression. Interestingly, this type of control can be achieved by either complete or partial degradation, but for a long time it was unclear how proteasomes designate ubiquitinated proteins for the specific type of degradation. Some light was shed with the discovery that Ter94, a homolog of CDC48/p97/VCP, and K11-linked ubiquitination regulate partial degradation in *Drosophila* [51]. This finding is particularly important since the Hedgehog family of proteins plays an evolutionarily conserved role during the development of metazoans, and disruption of Hedgehog signaling results in developmental disorders and cancers.

Qin et al. [52] succeeded in demonstrating that K11-linked ubiquitination can both prevent and promote the induction of type I IFN. This is achieved temporally as a result of a striking competition between K11- and K48-linked polyubiquitination of STING at K150. RNF26, an E3 ligase catalyzing the former type of ubiquitination in ER and mitochondria, protects STING from RNF5-mediated K48-linked polyubiquitination and its proteasomal degradation, leading to increased type I IFN and cytokine production [53]. At the same time, RNF26 limits excessive type I IFN production by induction of autophagic degradation of IRF3 [52], highlighting the cross-talk between autophagy and antiviral innate immunity.

In summary, APC/C- and other E3-mediated K11-linked ubiquitination of recently identified substrates, including NOXA [54], β‐TrCP1 [55], SOX [56], and Ci [57], earmark them for degradation. On the other hand, these chains can also modulate protein–protein interactions [58] or provide protein stabilization, as shown for β‐Catenin [59, 60]. Currently, it remains unclear whether these opposite effects arise from the action of different E3 ligases, different Ub-binding proteins acting as Ub receptors, or both. Nevertheless, UBE2S is the common denominator in all cases and remains the only E2 capable of promoting the K11-linked chain extension identified to date [37, 61].

Analogously to E3s, some USPs are engaged in the interplay between autophagy and immune responses. By removing K11-linked chains at K437 from Beclin-1, an essential factor of autophagic initiation, USP19 induces autophagy and inhibits the production of type I IFN [62]. Others reported modulation of the innate immune responses via K11-linked ubiquitination. There is ample evidence supporting the involvement of K11-linked ubiquitination in the control of the degradation of mitochondrial antiviral signaling protein (MAVS) after RNA virus infection [63] and degradation of cGAS once Zika virus NS1 protein recruits USP8 to remove K11-linked chains from caspase-1 [64]. USP8 has also been shown to preferentially remove K11-linked chains preventing the autophagic degradation of SQSTM1/p62 (sequestosome 1), a critical autophagy receptor that promotes the formation and degradation of ubiquitinated aggregates [65].

A number of studies have also shown a clear cleavage specificity for K11-linkages by the DUB named Cezanne [66–69]. This specificity provides post-translational as well as transcriptional control of cellular levels of hypoxia-inducible factors HIF1α and HIF2α. The former is stabilized by countering its K11 polyubiquitination, thus preventing its degradation through chaperone-mediated autophagy [70]; the latter, interestingly, is not directly targeted by Cezanne. Instead, Cezanne regulates the expression of HIF2α by controlling levels of its transcription factor, E2F1 [71]. Since the abundance of HIF-1α and HIF-2α proteins increases in many cancers, the control of their ubiquitination and proteasomal degradation may be of clinical importance. Such control could be achieved through the inhibition of specific DUBs. Indeed, DUBs have recently drawn much attention as therapeutic targets, as many are differentially expressed or activated in tumors. Nevertheless, the clinical development of selective DUB inhibitors faces multiple obstacles, with DUB specificity for Ub chains being one of them.

K11-linked conjugates represent the most abundant of the non-canonical Ub chain species in yeast [72, 73] and plants [74]. Early studies identified yeast Ubc6, an E2 involved in endoplasmic reticulum-associated degradation, as a K11-linked modified proteolytic substrate [73]. In plants, E2s play multiple roles in DNA repair, cellular responses to biotic and abiotic stresses, growth and development, as well as plant immunity. Interestingly, one of the E2s, UBC22, is more closely related to human UBE2S than to other E2s from *Arabidopsis thaliana* and mediates K11-linked ubiquitination [75]. Recently, UBC22 has been suggested to play a major role in many cellular processes [76], probably through its interactions with different E3s. Given that the number of E2s is roughly the same in human and *Arabidopsis* genomes (~ 40) [77] but E3s are about two and a half times more abundant in plants (~ 600 in the human genome but over 1,400 in *A. thaliana* [77]), one can assume that the UBC22-mediated K11-linked conjugation may be a much more common platform of the Ub landscape in plants than in humans.

## Lysine 27—a major player of innate immunity

Ubiquitination is a part of every step of viral infection [78] and K27-linked chains are heavily implicated in the regulation of NF-κB and IRF pathways. IRF3 serves both as a transcription factor for type I IFN production in the early phases of viral infection and a pro-apoptotic factor in the late phase of viral infection [79–82]. In zebrafish, both IRF3 and IRF7 can be decorated with K27-linked Ub chains to promote their proteasomal degradation [83]. Negative regulation of IRF3 has also been shown to be present in human cell lines. In this case, this regulation results from IRF3 autophagic degradation in a CALCOCO2/NDP52-dependent manner [84] that can be counteracted by PSMD14, a DUB which removes K27-linked chains from IRF3 and prevents its recognition by CALCOCO2 [84]. Thus, by promoting stabilization of IRF3, PSMD14 regulates type I IFN signaling and selective autophagy.

In the case of TBK1, at least three different modes of action are associated with K27-linked chains. Apart from the fact that they provide recruitment sites for TBK1 upon ubiquitination of STING, they also facilitate activation of TBK1 as a result of the migration of the K27-modified STING-TBK1 complex from ER membrane to perinuclear microsomes [85]. The STING-dependent signaling pathway is also positively regulated by RNF185, which enhances the enzymatic activity of cGAS through its K27-linked catalyzed polyubiquitination [86]. K27-linked chains are removed from STING by two DUBs, USP13 and USP21, inhibiting TBK1-dependent signal transduction and acting as negative regulators of the innate immune response [87, 88].

During dengue virus infection, NS3, one of the viral nonstructural proteins, interacts with NS2B to create the NS2B3 protease complex, which cleaves the viral polyprotein and some of the ER-localized proteins involved in the antiviral response, such as STING. It has been suggested that K27-linked ubiquitination of NS3 enhances protein–protein interactions and facilitates the formation of the NS2B3 complex [89]. However, this is a rather unexpected finding since this non-canonical ubiquitination has been previously shown to inhibit viral replication by promoting the proteasomal degradation of viral proteins from classical swine fever and porcine reproductive and respiratory syndrome viruses [90, 91]. Further experiments will be required to shed more light on K27-linked ubiquitination of viral proteins.

Proinflammatory cytokines, such as tumor necrosis factor α (TNFα), are widely known to activate TGFβ-activated kinase 1 (TAK1), which plays a significant role in the subsequent activation of NFκB signaling. To fine-tune its function, TAK1 undergoes multiple PTMs, including ubiquitination. While the role of K63- and K48-linked ubiquitination is widely discussed (reviewed in [92]), the scaffolding role of K27-linked ubiquitination has only recently been recognized in the case of TAK1. Once Ub chains are removed by USP19, the interaction between TAK1 and its partners TAB2 and TAB3 is abolished to downregulate TNFα- and IL-1β-induced NFκB activation [93]. Apart from its canonical role as an activator of NF-κB signaling, TNFα induces and sustains activation of MEK/ERK oncogenic signaling [94], where BRAF, a member of the RAF family protein kinases, is one of the key players [95]. Upon activation by inflammatory cytokines, the ITCH HECT-type E3 ligase ubiquitinates BRAF with K27-linked chains to provide sustained BRAF activation and to promote proliferation and invasion of melanoma cells [96]. Thus, one can imagine that ITCH could be targeted with therapeutic agents to prevent tumorigenesis, especially since ITCH has also been identified as an E3 targeting TIEG1 for K27-linked ubiquitination [97].

NEDD4 (neuronal precursor cell expressed, developmentally downregulated 4) and HACE1 (HECT domain and ankyrin repeat containing E3 ubiquitin ligase 1) are two E3 ligases known to promote K27-linked ubiquitination. The former E3 ligase is found to provide defense against bacterial infections through the stabilization of Beclin-1 with K6- and K27-linked ubiquitination [98]. The latter enzyme decorates optineurin, an autophagic receptor engaged in the disposal of damaged mitochondria [99], with K48- and K27-linked chains. The former type of Ub linkages leads to protein degradation, while the latter provides an interaction site between optineurin and SQSTM1 [100]. In all these cases, K27-linked ubiquitination is responsible for propelling autophagy progression. However, HACE1 can also ubiquitinate YB-1, a conserved DNA and RNA interacting factor whose function varies depending on its nuclear or cytoplasmic localization. Strikingly, K27-linked ubiquitination is required for secretion of YB-1 [101], but its extracellular functions are unknown.

The TRIM (Tripartite motif) family of E3 ligases carries out numerous tasks in the RLR-activated immune defense mechanism by assembling K27-linked chains and mediating protein–protein interactions. For instance, auto-ubiquitination of TRIM23 is required for its localization to autophagosomal membranes [102]. On the other hand, auto-ubiquitination of TRIM26 attracts NEMO and leads to type I IFN and cytokine production, as well as the expression of interferon-stimulated genes [103]. NEMO can itself become a target of K27-linked chains’ ubiquitination by TRIM23 [104]. In this case, Ub chains serve as a scaffold where the Rhbdd3 protease binds and recruits the DUB A20 via K27-linked chains attached to Rhbdd3. Once recruited, A20 deubiquitinates K63-linked polyUb chains on NEMO, suppressing TLR-triggered NF-κB activation in dendritic cells [105]. Another report also supports the role of K27-linked ubiquitination in controlling NF-κB as Hectd3 promotes K27- and K29-linked polyubiquitination on Malt1, and K27-linked polyubiquitination on Stat3, leading to NF-κB activation [106].

Extensive studies have shown that K27-linked ubiquitination negatively regulates cellular components involved in the immune response against RNA viruses. TRIM40 promotes the K27- and K48-linked polyubiquitination of MDA5 and RIG-I, leading to their degradation in the proteasome. This role in inhibiting IRF3 and activation of NF-kB has been suggested to prevent the pathogenesis of autoimmune diseases [107]. MAVS, which is recruited by MDA5 and RIG-I after sensing viral RNA, is ubiquitinated and designated for degradation. In human cells, this is accomplished by K27-linked chains on MAVS at K7 which serve as a recognition signal for autophagic degradation [108]. Conversely, K27-linked polyubiquitination of MAVS at K325 has been reported as a positive regulation signal for the innate immune response [109]. The above discrepancy may be explained by ubiquitination of different lysine residues and/or by implication of different E3 ligases: MARCH8 in the former case and TRIM21 in the latter case. However, further studies are required to elucidate the observed phenomena.

Similarly to K6 and K63-linked chains, K27 conjugates are also assembled onto H2A histones and provide binding platforms for DNA repair proteins such as 53BP1 [37, 110, 111]. A RING-type E3, RNF168, is responsible for assembling K27-linked polyUb-H2A conjugates [111]. It is postulated that K27 polyubiquitination is the major ubiquitination interlink induced by DNA damage [111]. On the other hand, the study above did not reveal significant participation of this kind of polymer during DDR [23]. Such a discrepancy might be ascribed to a different damaging agent used, etoposide in the former case [111] and ionizing radiation in the latter [23].

In addition to its role in antimicrobial and antiviral immunity, K27-linked ubiquitination has also been reported to play a role in anti-fungal signaling where TRIM62 activates CARD9 through its K27-linked ubiquitination [112]. It has also been observed that the joint presence of K27 and K29-linked chains promotes proteasomal and autophagic degradation, as well as protein aggregation associated with Parkinson’s disease (PD, see below) [113–115].

## Lysine 29—signaling and neurodegenerative disorders

As mentioned earlier, K29-linked ubiquitination of proteins has been implicated in neurodegenerative disorders. In the case of PD, TRAF6 promotes the aggregation of mutant DJ-1 and α-synuclein through K29-, but also K6-, and K27-linked chains [116]. In the pathogenesis of Huntington’s disease, the accumulation and aggregation of insoluble Ub-containing mHTT (the mutant huntingtin protein) have also been observed. These aggregates contain several E3 ligases, such as TRAF6, which has been found in the postmortem brains of Huntington’s disease patients and is decorated with K29-linked chains [117]. The sequestration of E3 ligases decreases the availability of active enzymes in the cell and abolishes their enzymatic activities, which may be important for recruiting their substrates into the aggregates. Remarkably, protein aggregation may constitute a protective mechanism against toxic proteins. Overexpression of the WSB1 E3 ligase counteracts LRRK2 neuronal toxicity [115], a common cause of PD. Neuronal protection of primary neurons is attributed to WSB1-catalyzed ubiquitination of LRRK2 with mixed K27/K29-linked chains, which stimulates aggregation presumably when K48-mediated proteasomal degradation of aberrant protein is no longer possible [115]. All in all, this effect justifies targeting WSB1 in the treatment of PD [118]. Taken together, these novel findings support the notion that atypical ubiquitination can exert opposite and regulatory effects in neurodegenerative disorders.

K29-linked chains have been reported to downregulate Wnt/β-catenin signaling [119, 120]. Smurf1-catalyzed ubiquitination of Axin with K29-linked chains blocks its interaction with the Wnt receptors and enables β-catenin disposal, stalling the Wnt pathway [120]. Smurf1 represses protein–protein interaction during autophagosome maturation, where it decorates UVRAG, a complex obligatory for this process, with K29 and K33-linked chains [121]. This leads to increased autophagic flux as modified UVRAG becomes unable to bind its inhibitor, Rubicon [122]. In contrast, UPS-mediated disposal of ULK1 modified with K27 and K29-linked Ub chains abolishes autophagic progression [114]. In this regard, K29-linked ubiquitination could be seen as the mechanism that controls autophagy during cellular stress.

The SKP1-Cullin-Fbx21 E3 ligase complex catalyzes K29-linked ubiquitination of Apoptosis signal-regulating kinase 1 (ASK1) [123]. This leads to the production of IFN β and interleukin 6, but details of this virus-induced activation of ASK1 signaling remain obscure. Another antiviral innate immune response involves K27- and K29-linked induced removal of MAVS aggregates by RNF34 [113]. Interestingly, only K27-linked modifications are obligatory for the autophagic intake in this case [113]. The same study also showed that RNF34 is an upregulator of mitophagy, leaving the question whether the removal of MAVS results from mitophagy or autophagy [113].

An interesting interplay is observed between Ufd2p and Ufd4, E2s found in *Saccharomyces cerevisiae*. The former catalyzes K48-linked multi-monoubiquitination of a substrate as long as the substrate carries K29-linked chains formed beforehand by the latter [124]. Even though this indicates that K29-linkage is a non-degradable signal that must be transformed into a proteasome-favored type, e.g., K48-linked chains, lysosomal degradation of DTX has been ascribed to the K29-linked ubiquitination mediated by ITCH/AIP4 [125].

Intriguingly, K29-linked ubiquitination can switch off malfunctioning proteasomes as shown in proteasome-inhibition proteomics studies [61]. During proteasomal stress, UBE3C catalyzes the assembly of K29-linked chains onto the Rpn13 proteasomal receptor [126]. A proteasome modified in such a manner loses its ability to bind ubiquitinated proteins [126].

Ubiquitination with K29/K33‐linked mixed chains has been shown to control AMP-activated protein kinases. While many of these enzymes remain largely uncharacterized, these kinases are considered to be involved in controlling whole-body energy homeostasis. Two members of this family, ARK5/NUAK1 and MARK4, exhibit K29/K33-linked polyubiquitination in vivo [127]. Importantly, the attachment of K29/K33‐coupled chains has been shown to interfere specifically with phosphorylation of the activation‐loop residues, thus blocking their kinase activity [127].

## Lysine 33—still much to learn

To date, K33-linked ubiquitination remains the least studied of all Ub linkage types. The first significant breakthrough came upon structural analysis of TRABID and its K29/K33-linkage specificity [128, 129]. However, there are still only very limited reports providing an in-depth explanation of the role of K33-linked ubiquitination in physiological processes. In some cases, this ubiquitination is identified but the functional consequences have not been explored [130]. This makes this type of linkage even more enigmatic, although there is a general consensus that K33-linked chains are likely non-degradative because this type of ubiquitination is not significantly enriched upon proteasomal inhibition [34].

One of the first reports came from the observation of increased T cell activation rates accompanied by spontaneous autoimmunity due to abnormal T cell receptor (TCR)-ζ chain phosphorylation in double knock-out mice cells lacking the Cbl-b and Itch E3 ligases [131]. This phosphorylation could be prevented by K33-linked ubiquitination at K54 of TCR. Therefore, the Ub-mediated regulation of TCR-ζ is not via degradation, but rather by modulation of interactions with its kinase, Zap70 [131].

The observed negative regulation of the innate immune responses via K33-linked ubiquitination may be explained by three possible mechanisms. One involves protein stabilization that has been reported in the case of TBK1 upon viral infection. That is to say, the removal of K33-linked chains from TBK1 by USP38 enables subsequent K48-linked ubiquitination and proteasomal degradation [132]. The other explanation arises from the fact that ubiquitination of the DNA-binding domain of STAT1, a type I interferon-induced transcription factor, inhibits STAT1 association with promoters of at least four interferon-stimulated genes and suppresses type I IFN-activated antiviral defense [133]. Lastly, the virus-induced translocation of IRF3 to the nucleus is impeded by K33-linked polyubiquitination within the IRF3 nuclear localization signal motif. More specifically, K33-linked ubiquitination disturbs protein–protein interactions between IRF3 and importin receptors [134] rather than its DNA-binding capabilities, thus preventing the translocation of IRF3 to the nucleus and expression of *IFNβ1* upon viral stimulation.

It has also been shown that K33-linked ubiquitination plays a significant role in protein trafficking. Coronin7 is a protein responsible for F-actin binding and stabilization during the synthesis of tubular-carrier precursors on the trans-Golgi network (TGN). KLHL20-mediated non-degradable K33-linked ubiquitination of Coronin7 promotes its interaction with Eps15, a UBD-containing clathrin adaptor required for post-Golgi trafficking. The subsequent recruitment of Coronin7 to TGN induces the biogenesis of TGN-derived transport carriers [135]. Therefore, K33- is the latest addition to the Ub linkages, mainly K63-, which may serve as protein sorting signals.

Emerging in vivo imaging methods revealed the co-localization of K33-linked polyUb chains and Ub-binding autophagy adaptors, such as LC3B and its interacting partner SQSTM1 [136]. Indeed, SQSTM1 was later shown to be decorated with K29- and K33-linked chains in antibacterial autophagy [137]. The most recent findings connect LZTR1-mediated K33-linked ubiquitination of RAS-GTPases and autophagy via their interactions with SQSTM1 [138]. Notably, LZTR1 had been previously implicated in autophagy through its colocalization with LC3B [139]. In conclusion, these discoveries strongly interlink K33 ubiquitination and autophagy, although the precise role of this atypical ubiquitination remains elusive.

## Summary and future outlook

The deciphering of the Ub code has continued to advance significantly in recent years.. The latest biochemical and Ub-enrichment methods, as well as mass spectrometry-based approaches, enabled us to study the variety of Ub chains and their unique properties at an unprecedented level (Table 1). This rapid development is often driven by the studies of dysfunction of Ub signaling pathways as they offer hope for targeted therapies in cancer [140] and neurodegenerative disorders [141–143].

**Table 1:** Overview of non-K48/K63-linked ubiquitination

Process/effect	Ub linkage(s)	USP/DUB	E2(s)	E3(s)	Substrate(s)
Mitophagy	K6/K11/K48/K63	USP30		Parkin	[12] [14]
Mitophagy	K6	USP30			TOM20 [19]
DDR	K6			BRCA1-BARD1	BRCA1-BARD1 [21]
DDR	K6			BRCA1-BARD1	[22]
DDR	K6			RNF8	Nbs1 [26]
DDR	K6/K48/K63			HUWE1	Chk1 [25]
Many	K6			HUWE1	VCP [24, 27]
D/S	K6			HUWE1	Mfn2 [27]
n.d	K6/K11/K48/K63		UBCH7/UBE2L3	RNF144A, RNF144B	[27]
D/S	K6			MGRN1	α-Tubulin [31, 32]
D/S	K6/K11			CBLC	EGFR [33]
D/S	K6			HUWE1	Ubl4A [28]
D/S	K6			UBE4A	Viperin [29]
DNA binding	K6/K11/K29	OTUD1			IRF3 [36]
DDR	K11		UBE2S	RNF8	H2A/H2AX [37]
D/S	K11		UBE2F	SAG-CUL5	NOXA [54]
D/S	K11		UBE2S	SAG/RBX2	β-TrCP1 [55]
D/S	K11		UBE2S		β-Catenin [60]
D/S	K11/ K29			EDD	β-Catenin [59]
D/S	K11		UBE2S		SOX [56]
PPI	K11		UBCH5	c-IAP1	RIP1 [58]
D/S	K11		UbcD1	Cul1-Slimb	Ci [51, 57]
D/S	K11		UBE2S, UBCH10	APC/C	Nek2A [40]; Bard1, Hmmr, HURP, NuSAP [42, 43, 45]
D/S	K11	Cezanne	UBE2S	APC/C	Cyclin B, Securin [69]
D/S	K11	Cezanne			HIF1α [70]
D/S	K11	Cezanne			E2F1 [71]
D/S	K11	USP19			HIF1α [147]
Innate immunity	K11	USP19			Beclin-1 [62]
D/S	K11	USP8			Caspase-1 [64]
D/S	K11	USP8			SQSTM1 [65]
Innate immunity	K11			RNF26	STING [52]
D/S	K11/K48			UBR5	TIP60 [49]
D/S	K11/K48	RAD6		KCMF1-UBR4	[48]
D/S	K11/K48			SKP1-Cullin-Fbx21	Ci [51]
Plant development/ stress response	K11	UBC22			[75, 75]
PPI	K27			RNF168	H2A [111]
D/S	K27			fbxo3	IRF3, IRF7 [83]
PPI	K27	PSMD14			IRF3 [84]
Innate immunity	K27			AMFR	STING [85]
Innate immunity	K27	USP13, USP21			STING [87, 88]
Innate immunity	K27			RNF185	cGAS [86]
Innate immunity	K27	USP19			TAK1 [93]
Protein activation	K27			ITCH	BRAF [96]
Localization	K27			ITCH	TIEG1 [97]
D/S	K27/K6			NEDD4	Beclin-1 [98]
PPI	K27/K48			HACE1	Optineurin [99]
PPI	K27			HACE1	YB-1 [101]
Innate immunity	K27			TRIM23	NEMO [104]
Innate immunity	K27				Rhbdd3, NEMO [105]
Autophagy	K27			TRIM23	TRIM23 [102]
Innate immunity	K27			TRIM26	TRIM26 [103]
Protein activity	K27, K27/K29			Hectd3 Hectd3	Stat3, Malt1 [106]
Innate immunity	K27			TRIM40	RIG-I, MDA5 [107]
Innate immunity	K27			MARCH8	MAVS [108]
Innate immunity	K27			TRIM21	MAVS [109]
PPI	K27				NS3 [89]
D/S	K27			RNF114	NS4B [90] Nsp12 [91]
Protein activity	K27			TRIM62	CARD9 [112]
Aggregation	K27/K29			WSB1	LRRK2 [115]
Aggregation	K29/K6/K27			TRAF6	HTT [116]
Protein activity	K29			Trim13	TRAF6 [117]
PPI	K29, K29/K33			Smurf1 Smurf1	Axin [120] UVRAG [121]
Innate immunity	K29			SKP1-Cullin-Fbx21	ASK1 [123]
Autophagy	K27/K29			RNF34	MAVS [113]
D/S	K29/K48		Ubc4p	Ufd4p	[124]
D/S	K29			ITCH/AIP4	DTX [125]
Protein activity	K29		UBE3C		Rpn13 [126]
Protein activity	K29/K33	USP9X			ARK5/NUAK1, MARK4 [127]
Signaling	K33			Cbl-b, Itch	TCR-ζ [131]
Innate immunity	K33	USP38			TBK1 [132]
Innate immunity	K33			RNF2	STAT1 [133]
	K33			RNF152	RagA [130]
Trafficking	K33			KLHL20	Coronin7 [135]
D/S	K33			LZTR1	RAS [138]
Localization	K33				LC3B, SQSTM1 [136]
Autophagy	K29, K33	ZRANB1		RNF166	SQSTM1 [128, 129] [137]

*D/S* protein degradation or stabilization, *n.d.* not determined, *PPI* protein–protein interactions

A number of questions regarding ubiquitination remain to be addressed, including the possibility of non-protein ubiquitination [144] or the co-existence of many Ub combinations in a cell at any given time. In the latter case, it has already been shown that the N-terminus of proteins, cysteine, serine, and threonine residues can all serve as sites for ubiquitination [145]. In addition, other PTMs, such as acetylation, deamidation, phosphorylation, or ADP-ribosylation of Ub, have also been reported, adding yet another layer of Ub complexity [146]. Overall, studying the Ub code, proteins and enzymes involved in (de)ubiquitination pathways offers the possibility for the insightful understanding of the majority of cellular processes.

## Abbreviations

APC/C: Anaphase promoting complex/cyclosome
DDR: DNA damage response
DUB: Deubiquitinase
HACE1: HECT domain and ankyrin repeat containing E3 ubiquitin ligase 1
HECT: Homologous to the E6-AP C-terminus
HIF: Hypoxia-inducible factor
HUWE1: HECT, UBA and WWE domain-containing protein 1
IFN: Interferon
IRF3: Interferon regulatory factor 3
MARCH: Membrane-associated E3 ubiquitin ligase containing RING-CH-type zinc finger motifs
MAVS: Mitochondrial antiviral signaling
NEDD4: Neuronal precursor cell expressed, developmentally downregulated 4
NEMO: NF-kappaB essential modulator
OMM: Outer mitochondrial membrane
PD: Parkinson’s disease
PINK1: PTEN-induced kinase 1
PPI: Protein–protein interactions
PTM: Post-translational modification
RING: Really interesting new gene
RLR: RIG-I-like receptor
RNF: RING finger protein
STING: Stimulator of interferon genes
TBK1: TANK-binding kinase 1
TCR: T cell receptor
TLR: Toll-like receptor
TNFα: Tumor necrosis factor α
TRIM: Tripartite motif-containing protein
Ub: Ubiquitin
UBD: Ubiquitin-binding domain
UBR: Ubiquitin ligase E3 component N-recognin
UPS: Ubiquitin/proteasome system
USP: Ubiquitin-specific proteases
